# 1,25-Dihydroxyvitamin D to PTH(1–84) Ratios Strongly Predict Cardiovascular Death in Heart Failure

**DOI:** 10.1371/journal.pone.0135427

**Published:** 2015-08-26

**Authors:** Damien Gruson, Benjamin Ferracin, Sylvie A. Ahn, Claudia Zierold, Frank Blocki, Douglas M. Hawkins, Fabrizio Bonelli, Michel F. Rousseau

**Affiliations:** 1 Pôle de recherche en Endocrinologie, Diabète et Nutrition, Institut de Recherche Expérimentale et Clinique, Cliniques Universitaires St-Luc and Université Catholique de Louvain, Brussels, Belgium; 2 Department of Laboratory Medicine, Cliniques Universitaires St-Luc and Université Catholique de Louvain, Brussels, Belgium; 3 Division of Cardiology, Cliniques Universitaires St-Luc and Pôle de recherche cardiovasculaire, Institut de Recherche Expérimentale et Clinique, Université Catholique de Louvain, Brussels, Belgium; 4 DiaSorin Inc, 1951 Northwestern Avenue, Stillwater, Minnesota, 55082, United States of America; 5 School of Statistics, University of Minnesota, Minneapolis, Minnesota, 55455, United States of America; Fondazione G. Monasterio, ITALY

## Abstract

**Objectives:**

Vitamin D deficiency and hyperparathyroidism are common in patients with heart failure (HF). There is a growing body of evidence supporting the role of vitamin D and parathyroid hormone (PTH) in cardiac remodeling and worsening of HF. Lack of reliable automated testing of 1,25-dihydroxyvitamin D (1,25(OH)_2_D), the biologically active metabolite of vitamin D, has limited its contribution to the prognostic assessment of HF. Here, the association of 1,25(OH)_2_D and PTH(1–84) levels was evaluated for prediction of cardiovascular death in chronic HF patients.

**Methods:**

We conducted a single center prospective cohort including 170 chronic HF patients (females n = 36; males n = 134; NYHA II-IV; mean age: 67 years; etiology: ischemic n = 119, dilated cardiomyopathy n = 51; mean LVEF: 23%). The primary outcome was cardiovascular death.

**Results:**

Serum levels of 1,25(OH)_2_D decreased markedly with increased HF severity. Medians were 33.3 pg/mL for NYHA-II patients, 23.4 pg/mL for NYHA-III, and 14.0 pg/mL for NYHA-IV patients (p<0.001). Most patients had levels of 25(OH)D below 30ng/mL, and stratification by NYHA functional class did not show significant differences (p = 0.249). The 1,25(OH)_2_D to PTH(1–84) ratio and the (1,25(OH)_2_D)^2^ to PTH(1–84) ratio were found to be the most significantly related to HF severity. After a median follow-up of 4.1 years, 106 out of 170 patients reached the primary endpoint. Cox proportional hazard modeling revealed 1,25(OH)_2_D and the 1,25(OH)_2_D to PTH(1–84) ratios to be strongly predictive of outcomes.

**Conclusions:**

1,25(OH)_2_D and its ratios to PTH(1–84) strongly and independently predict cardiovascular mortality in chronic HF.

## Background

Cardiovascular (CV) diseases remain a leading cause of death around the world [1]. Among CV diseases, heart failure (HF) represents a major health concern because of increasing prevalence worldwide with major human, societal and economic impacts [2–7]. The need for biomarkers for the prognosis of HF is well established, and different biomarkers from several pathophysiological pathways have been evaluated in this setting [8–13].

There is a growing body of evidence supporting the role of vitamin D and parathyroid hormone (PTH) in cardiac remodeling and worsening HF [14–17]. Furthermore, PTH together with aldosterone and fibroblast growth factor 23 (FGF-23), may also be part of a vicious and deleterious cycle which compromises CV function [18]. Markedly elevated levels of FGF-23 and PTH were observed in patients with CV disorders and HF, and have been related to adverse CV events [14;15;19–21].

Like PTH and FGF-23, 1,25-dihydroxyvitamin D (1,25(OH)_2_D, calcitriol) is an important regulator of calcium and phosphate homeostasis [21–23]. Recently, a novel fully-automated 1,25(OH)_2_D assay with improved analytical performance, sensitivity, and reliability has emerged [22;24]. The imprecision at low levels of existing 1,25(OH)_2_D measurement has precluded the ability to identify meaningful clinical correlates of HF progression so far. The aim of this study, therefore, was to assess the impact of sensitive, precise, accurate 1,25(OH)_2_D measurement and its ratios to PTH(1–84) on CV survival in HF patients.

## Methods

### Study population

We prospectively assessed CV death of 170 consecutive fully treated patients with chronic HF and reduced left ventricular ejection fraction (LVEF) followed at the Cliniques Universitaires Saint-Luc, an academic hospital of Brussels, Belgium, between March 30^th^ 2004 and June 16^th^ 2006. Patients with left ventricular systolic dysfunction and ejection fraction of 35% or less were eligible for the study. Ejection fraction was measured by radionuclide technique or contrast ventriculography, the latter being associated with coronary arteriography to confirm ischemic etiology. Exclusion criteria were age <18 years, LVEF higher than 35%, abnormal liver function test (AST/ALT 2 times the upper limit of the reference interval), anaemia or iron reserve deficiencies, genetic hypertrophic cardiopathy, severe pulmonary diseases (COPD gold 3–4), patients under dialysis and primary hyperparathyroidism. Survival status was obtained by phone contact with patients, their relatives, or their physicians.

### Ethics statement

The research protocol conformed to the ethical guidelines of the 1975 Declaration of Helsinki and all participants gave verbal informed consent regarding the goals of the study and their willingness to participate. The ethics committee of the Catholic University of Louvain approved this study as well as the consent procedure.

### Clinical outcomes

Patient history and treatment was retrieved from medical files and review of hospital visitation records. Follow-up events including CV mortality and cardiac transplantation were 100% complete. Cardiac death was defined as death attributable to congestive HF, myocardial infarction, sudden death, or death occurring pursuant to revascularization procedures.

### Laboratory measurements

Routine laboratory measurements and blood samples for biomarker analyses were obtained at hospital admission. Venous blood samples were obtained at enrollment, processed, and stored at -80°C until time of assay. Levels of 1,25(OH)_2_D were determined at baseline with a fully automated and sensitive immunoassay that uses a recombinant fusion construct of the vitamin D receptor ligand binding domain for specific capture of 1,25(OH)_2_D (DiaSorin, Saluggia, Italy). The limit of quantitation for this 1,25(OH)_2_D assay is 5 pg/mL and the reference interval determined in healthy volunteers ranged between 25.0 and 86.5 pg/mL with a median of 48.1 pg/mL. Plasma Ct-FGF23 concentrations were determined with a second-generation C-terminal human enzyme-linked immunosorbent assay (Immutopics, San Clemente, CA, USA). Levels of 25(OH)D, PTH(1–84) (both DiaSorin), B-type natriuretic peptide (BNP, Beckman Coulter, Fullerton, CA, USA; Alere reagents), N-terminal proBNP (NT-proBNP, Roche Diagnostics, Mannheim, Germany), Chromogranin A (CgA, Dako, Glostrup, Denmark), and Galectin-3 (Gal-3, BG Medicine, Waltham, MA, USA) were also determined. Glomerular filtration rate (eGFR) was estimated by the Modification of Diet in Renal Disease formula.

### Statistical analysis

Independent determinants of baseline 1,25(OH)_2_D levels were assessed using multivariable linear regression methods with Log-transformed levels of 1,25(OH)_2_D as the dependent variable. The discriminatory power between biomarkers was assessed by fitting Cox proportional hazard (CPH) models using each of them, along with patient characteristics of age, sex, clinical variables, etiology of the disease, previous admission to hospital and treatment to predict outcomes. Biomarkers with highly skewed distributions – 1,25(OH)_2_D, PTH(1–84), NT-proBNP, 25(OH)D, FGF-23, and creatinine were Log-transformed for use in the CPH models. The strength of association of each of these predictors can be summarized by the chi-squared statistic of the fitted CPH. Along with the individual markers and patient characteristics, 1,25(OH)_2_D to PTH(1–84) ratios were also determined.

Several of these predictors have large chi-squared values, indicating strong associations with survival. For these biomarkers, receiver operating curve (ROC) analyses were performed to explore the trade-off between sensitivity and specificity as assessed at the seven-year mark, and to locate a cut point suitable for use in dichotomizing the patients into high- and low-risk groups. This cut point was defined as that value maximizing the difference between sensitivity and false positive rates. The Kaplan-Meier survival curve of patients split by this dichotomy was found and tested using the Log-Rank test. P-values < 0.05 were considered significant. Statistical analysis was performed using R version 3.1.0 (The R Foundation for Statistical Computing) and JMP software version 11.00 (SAS institute, NC, USA). It may be mentioned that the CPH and the ROC analyses involve somewhat different perspectives. The ROC calculations use a snapshot of the patients at the end of the monitoring period and do not distinguish between earlier and later deaths. The CPH and Kaplan-Meier analyses, on the other hand, use the entire survival function and thereby do distinguish earlier from later deaths.

## Results

### Baseline characteristics

Table 1 shows the baseline characteristics and laboratory values of the study population, according to 25(OH)D and 1,25(OH)_2_D levels. 170 chronic HF patients were included (mean age 67±14 years; females n = 36; males n = 134; New York Heart Association (NYHA) II-IV; etiology: ischemic n = 119, dilated cardiomyopathy n = 51; mean LVEF 23±7%).

**Table 1:** Baseline characteristics and biomarkers of the study population according to median levels of 25(OH)D and 1,25(OH)_2_D.

Characteristics	Entire cohort	25(OH)D less than median	25(OH)D greater than median	P-value	1,25(OH)_2_D less than median	1,25(OH)_2_D greater than median	P-value
**Age (years)**	69 [21–89]	66 [33–89]	70 [21–87]	0.559	70 [33–89]	67 [21–86]	0.116
**Sex (M/F)**	134/36	64/21	70/15	0.808	67/18	67/18	0.785
**Dilated cardiomyopathy (%)**	30	26	34	0.016	26	34	0.016
**Ischemic cardiomyopathy (%)**	70	74	66	0.022	74	66	0.022
**EF (%)**	24 [8–35]	23 [8–35]	24 [9–35]	0.119	23 [9–35]	23 [8–35]	0.970
**Heart Rate**	79 [46–135]	79 [50–135]	78 [46–124]	0.893	79 [51–135]	78 [46–130]	0.795
**Diabetes (%)**	29	31	26	0.095	38	20	0.038
**Hypertension (%)**	55	49	61	0.031	45	55	0.047
**Previous admission to hospital (%)**	32	27	34	0.045	38	41	0.112
**Smoker C/F/N (%)**	16/25/59	21/20/59	12/31/57	0.108	15/27/58	18/24/58	0.467
**Treatment**							
**ACE inhibitors (%)**	79	70	85	0.037	76	77	0.324
**β-blockers (%)**	83	83	81	0.134	86	80	0.112
**Diuretics (%)**	72	76	74	0.250	85	64	<0.001
**Aldosterone antagonists (%)**	62	60	75	0.027	73	61	0.030
**Angiotensin II receptor blockers (%)**	20	25	17	0.017	26	18	0.023
**Vitamin D (%)**	3	2	4	0.346	5	1	0.076
**Anticoagulant (%)**	38	40	35	0.332	41	35	0.276
**Antiplatelet (%)**	67	73	60	0.030	68	66	0.649
**Antidiabetic drug (%)**	28	30	25	0.112	36	20	0.024
**eGFR (mL/min/1.73m** ^**2**^ **)**	56.1 [9.4–144.6]	55.6 [9.4–144.6]	58.5 [11.0–114.7]	0.958	51.6 [9.4–107.8]	61.6 [22.2–144.6]	0.001
**Calcium, total (mg/dL)**	8.9 [6.2–10.8]	8.9 [6.2–10.8]	8.9 [7.8–10.4]	0.320	8.8 [6.2–10.4]	9.0 [7.8–10.8]	0.007
**LDL-Cholesterol (mg/dL)**	97 [16–220]	99 [16–185]	97 [32–220]	0.434	91 [16–180]	100 [32–220]	0.459
**Triglycerides (mg/dL)**	90 [14–345]	90 [14–345]	91 [28–282]	0.522	85 [14–231]	99 [39–345]	0.256
**25(OH)D (ng/mL)**	12.3 [4.3–46.1]	8.5 [4.3–12.1]	18.8 [12.5–46.1]	< 0.001	10.9 [4.3–34.0]	15.0 [5.0–46.1]	0.024
**1,25(OH)**_**2**_ **D (pg/mL)**	25.4 [5.0–100]	22.5 [5.0–74.1]	28.4 [7.5–100]	0.025	17.4 [5.0–25.2]	35.8 [25.6–100]	<0.001
**PTH 1–84 (pg/mL)**	45 [4–244]	52 [12–244]	37 [4–201]	<0.001	44 [8.7–244]	45 [4–201]	0.024
**BNP (ng/L)**	455 [17–5017]	756 [21–5017]	308 [17–4408]	<0.001	687 [43–5017]	299 [17–4408]	<0.001
**Nt-proBNP (ng/L)**	2157 [66–33020]	3385 [66–33020]	1571 [95–21295]	<0.001	3276 [71–33020]	929 [66–29925]	<0.001
**CgA (UI/L)**	35.1 [4.9–422]	34.8 [6.8–384]	34.4 [4.9–422]	0.889	46.8 [7.9–422]	26.7 [4.9–384]	< 0.001
**Gal-3 (ng/mL)**	18.0 [7.8–49.6]	18.0 [7.9–45.5]	17.4 [9.8–49.6]	0.453	20.1 [9.9–49.6]	14.9 [7.9–45.5]	< 0.001
**FGF-23 (RU/mL)**	1126 [23–15000]	228 [23–13906]	148 [32–15000]	<0.001	300 [32–15000]	101 [23–10301]	<0.001
**1,25(OH)**_**2**_ **D/PTH(1–84)**	0.62 [0.02–9.33]	0.45 [0.02–3.76]	0.89 [0.12–9.33]	<0.001	0.38 [0.02–2.84]	1.15 [0.15–9.33]	<0.001
**(1,25(OH)**_**2**_ **D)** ^**2**^ **/PTH(1–84)**	15.3 [0.10–357]	9.7 [0.10–248]	23.7 [1.6–357]	<0.001	6.5 [0.10–70.1]	45.5 [4.8–357]	<0.001

Most patients had levels of 25(OH)D below 30 ng/mL (58% of HF patients were below 15 ng/mL, 21% between 15 and 20 ng/mL, 15% between 20 and 30 ng/mL, and only 6% were higher than 30 ng/mL, and stratification by NYHA functional class did not disclose significant differences (p = 0.249). In contrast, median serum levels of 1,25(OH)_2_D decreased markedly according to HF severity: 33.3 pg/mL in NYHA class II (n = 60), 23.4 pg/mL in NYHA class III (n = 94), and 14.0 pg/mL in NYHA class IV (n = 16; p<0.001). The median 1,25(OH)_2_D level for all patients was 25.4 pg/mL (range: 5.0–100.0 pg/mL). Participants with 1,25(OH)_2_D levels below the median 25.4 pg/mL value were more likely to have higher circulating levels of BNP, NT-proBNP, FGF-23, CgA and Galectin-3.

The median PTH(1–84) levels by severity were as follows: NYHA class II 27.6 pg/mL, NYHA class III 41.4 pg/mL, and NYHA class IV 41.0 pg/mL (p<0.01 between Class II and III). The median for PTH(1–84) for all patients was 38.0 pg/mL (range: 4.0–244 pg/mL). Because 1,25(OH)_2_D and PTH(1–84) are physiologically interrelated (23), we examined the ratio of these two hormones and found them to be significantly related to HF severity: NYHA class II ratio = 1.14, NYHA class III ratio = 0.47, and NYHA class IV ratio = 0.38 (p<0.001). The median ratio for all patients was 0.62 (range: 0.02–9.3). The 1,25(OH)_2_D to PTH(1–84) ratios were higher in patients with 1,25(OH)_2_D levels above the median, while PTH was not different between the two groups.

In multiple regression analyses including age, LVEF, clinical variables, eGFR, 1,25(OH)_2_D, PTH(1–84), NT-proBNP, 25(OH)D, FGF-23, CgA and Gal-3, the independent determinants of baseline 1,25(OH)_2_D were age, eGFR, PTH(1–84), and Galectin-3. The levels of 1,25(OH)_2_D were not significantly different between patients with an ischemic origin of the disease (26.6 pg/mL) in comparison to dilated cardiopathy (31.5 pg/mL; p = 0.057). The 1,25(OH)_2_D concentrations were not significantly different between patient receiving β-blockers (28.0 pg/mL) in comparison to those not receiving β-blockers (28.1 pg/mL; p = 0.981). Levels of 1,25(OH)_2_D were correlated to eGFR (ρ = 0.42, p<0.0001). In HF patients with normal kidney function (eGFR>60 mL/min/1.73m^2^, n = 74), the median 1,25(OH)_2_D level was 31.5 pg/mL, which remains clearly lower than the median of healthy individuals (49.6 pg/mL, data not shown); in this group of patients the 1,25(OH)_2_D to PTH(1–84) ratios remain significantly correlated to BNP, NT-proBNP, CgA, Gal-3 and FGF-23, while 1,25(OH)_2_D is not significantly correlated to NT-pro-BNP in patients with eGFR>60 mL/min/1.73m^2^. HF patients with a history of previous admission to hospital have higher levels of 1,25(OH)_2_D (29.7 pg/mL) in comparison to those without previous admission to hospital wards (24.4 pg/mL; p = 0.037).

### 1,25(OH)_2_D level and 1,25(OH)_2_D to PTH(1–84) ratio outcomes in chronic HF

Over a median follow-up time of 4.1 years (occurrence of a first endpoint and maximum follow-up times were 7 days and 7.4 years, respectively), 106 HF patients met the endpoint of which 94 died, and 12 underwent heart transplant. Biomarker concentrations as well as clinical variables between patients that developed the outcome and those who remained stable are presented in Table 2.

**Table 2:** Clinical variables and biomarkers among stable HF patients and HF patients who developed the outcome.

Variables	Stable patients (n = 64)	Patients with outcomes (n = 106)	P-value
**Age (years)**	60.4	68.6	0.001
**Sex (M/F)**	47/17	87/19	0.153
**Dilated cardiomyopathy (%)**	42	22	0.046
**Ischemic cardiomyopathy (%)**	58	78	0.042
**EF (%)**	23.7	20.3	0.003
**Heart Rate**	79	79	0.897
**Diabetes (%)**	28.8	37.6	0.303
**Hypertension (%)**	55.3	62.4	0.358
**Previous admission to hospital (%)**	31.8	62.4	<0.001
**Treatment**			
**ACE inhibitors (%)**	73	77	0.203
**β-blockers (%)**	63.0	77.8	0.048
**Diuretics (%)**	63.0	75.6	0.076
**Aldosterone antagonists (%)**	63.0	64.4	0.521
**Angiotensin II receptor blockers (%)**	20.7	24.0	0.655
**Vitamin D (%)**	1	3	0.487
**Anticoagulant (%)**	37.6	38.2	0.196
**Antiplatelet (%)**	67.1	62.4	0.633
**Antidiabetic drug (%)**	28.2	37.6	0.366
**eGFR (mL/min/1.73m** ^**2**^ **)**	61.7	49.2	0.002
**Calcium, total (mg/dL)**	8.9	8.8	0.955
**BNP (ng/L)**	227	666	< 0.001
**NT-proBNP (ng/L)**	1012	2992	< 0.001
**Gal-3 (ng/mL)**	16.3	19.5	0.001
**CgA (UI/L)**	26.6	47.2	< 0.001
**FGF-23 (RU/mL)**	149	413	< 0.001
**PTH (1–84)(pg/mL)**	28	41	0.001
**25(OH)D (ng/mL)**	13.1	12.4	0.488
**1,25(OH)**_**2**_ **D (pg/mL)**	31.7	20.2	< 0.001
**1,25(OH)**_**2**_ **D / PTH(1–84)**	1.12	0.49	< 0.001
**(1,25(OH)**_**2**_ **D)** ^**2**^ **/ PTH(1–84)**	35.38	9.91	< 0.001

CPH analysis predicting survival from the Logs of 1,25(OH)_2_D and PTH(1–84) show that each of these biomarkers is highly significant even when the other is used (their p-values in the CPH using both being 1.2x10^-7^ and 1.5x10^-3^); in other words, both contribute separate, highly significant, predictive power. The coefficient of Log 1,25(OH)_2_D is roughly -2 times that of Log PTH(1–84), suggesting that the two might be summarized by a score function -2 Log 1,25(OH)_2_D + Log PTH(1–84), which is the negative Log of the ratio of the square of the 1,25(OH)_2_D assay to the PTH. This composite score marker is assessed in Table 3 as “Square ratio”, and indeed it has the second-highest chi-squared value of all the biomarkers, being outperformed only (and very modestly) by BNP. A simpler approach using the 1,25(OH)_2_D to PTH(1–84) ratio, without the doubling on the 1,25(OH)_2_D, is assessed under the label “Ratio”. As Table 3 might lead one to expect, this simpler ratio does not perform quite as well, but nevertheless is beaten only by BNP and NT-proBNP. Thus both the simple 1,25(OH)_2_D/PTH(1–84) ratio and the slightly more complex (1,25(OH)_2_D)^2^/PTH(1–84) ratio are highly competitive risk scores.

**Table 3:** Strength of biomarkers determined by CPH fits and respective chi-square.

Biomarker	Chi-square
BNP	39.2
Square ratio	38.9
NT-proBNP	37.3
Ratio	36.0
FGF-23	35.0
NYHA	31.0
1,25(OH)_2_D	29.1
Galectin-3	15.6
LVEF	13.8
PTH(1–84)	12.3
Age	10.7
Creatinin	9.1
Etiology	8.3
Sex	1.1
Calcium, total	1.0

The biomarkers with highly skewed distributions were Log-transformed (Square ratio, Ratio, NT-proBNP, 1,25(OH)_2_D, PTH(1–84), FGF-23, and creatinine). Square ratio = (1,25(OH)_2_D)^2^/PTH(1–84), Ratio = 1,25(OH)_2_D/PTH(1–84).

**Fig 1:**
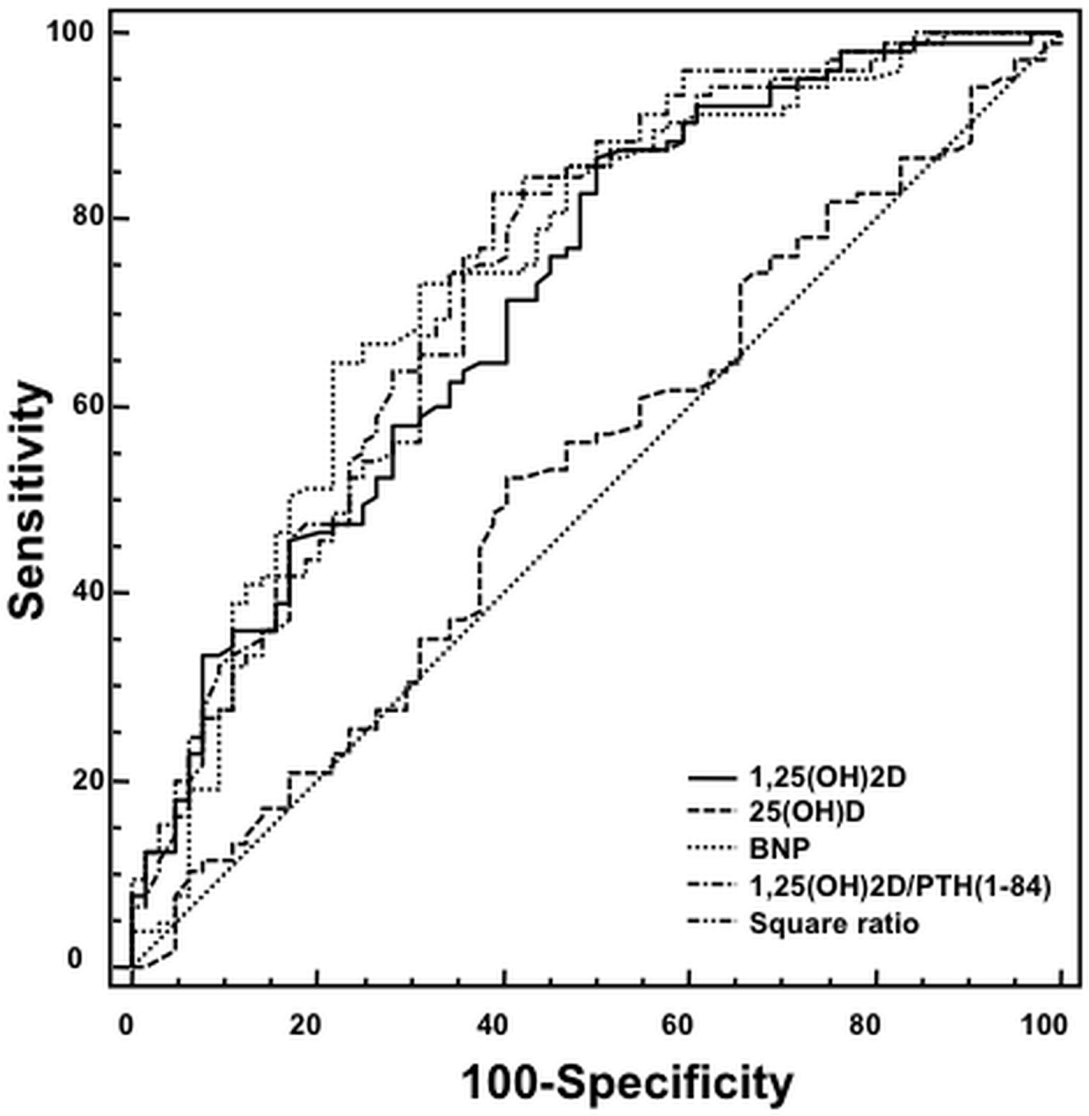
Receiver operating characteristic (ROC) analysis was performed for CV death at the end of the follow-up for 25(OH)D, BNP, 1,25(OH)_2_D and its ratios to PTH(1–84).

Going from the CPH to the ROC approach and focusing just on the seven-year survival, in ROC analysis the area under the curve (AUC), criteria defined as CV death at the end of the follow-up, for 1,25(OH)_2_D and the ratios of 1,25(OH)_2_D/PTH(1–84) and (1,25(OH)_2_D)^2^/PTH(1–84) were 0.722 (95% CI: 0.648–0.788), 0.741 (95% CI: 0.668–0.805), and 0.749 (95% CI: 0.677–0.812) respectively, which was similar to BNP (AUC 0.744 [(95% CI: 0.671–0.808]), but clearly higher than 25(OH)D (AUC 0.529 [(95% CI: 0.451–0.606]; p<0.01) (Fig 1). Clinically accepted biomarkers had AUCs as follows: NT-proBNP (AUC 0.730 [(95% CI: 0.657–0.796]), Gal-3 (AUC 0.660 [(95% CI: 0.583–0.731]), FGF-23 (AUC 0.702 [(95% CI: 0.624–0.773]), CgA (AUC 0.663 [(95% CI: 0.587–0.733]), and PTH(1–84) (AUC 0.641 [(95% CI: 0.564–0.713]).

**Fig 2:**
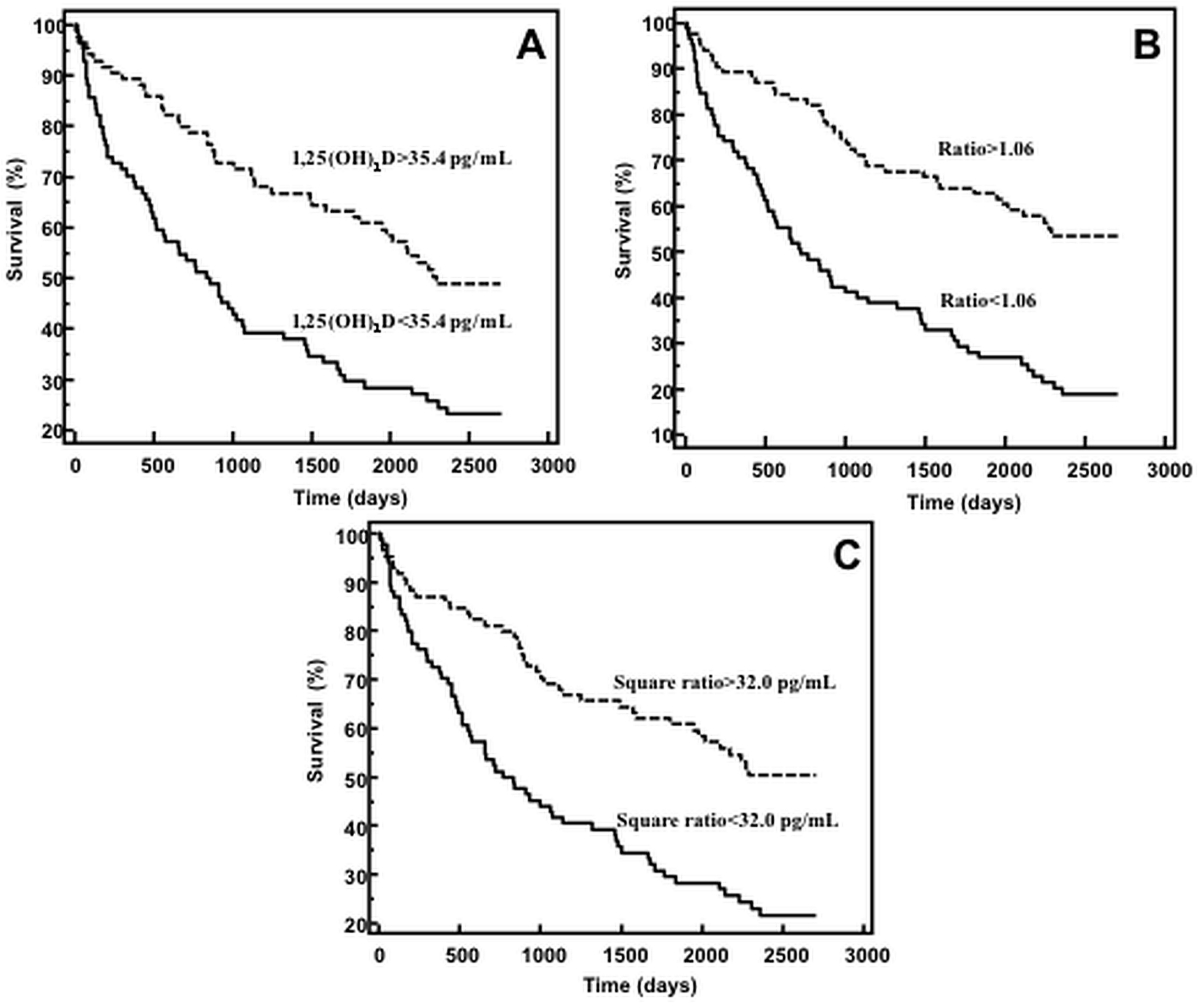
Kaplan-Meier survival curves stratified by (A) 1,25(OH)_2_D levels, (B) the ratio of 1,25(OH)_2_D to PTH(1–84) (Ratio), and (C) the ratio of (1,25(OH)_2_D)^2^ to PTH(1–84) (Square ratio).

Youden’s index and the associated criterion based on the ROC curve analysis were used for the stratification of the Kaplan-Meier curves (35.4 pg/mL for 1,25(OH)_2_D, 1.06 for the ratio, 32.0 pg/mL for the Square ratio). Kaplan Meier survival curves for patients stratified by 1,25(OH)_2_D levels diverged significantly (Log-rank test: p <0.001; Fig 2A). Survival curves for patients stratified by the 1,25(OH)_2_D to PTH(1–84) ratios also diverged significantly (Log-rank test: p <0.001; Fig 2B and 2C).

Table 4 shows the coefficients and the statistical significance of each term in a multivariate CPH model including all the variables listed in the table. In this table, the contribution of each predictor is adjusted for the common predictive information of all other predictors. The Square ratio has a highly significant p-value (0.011) showing that this predictor is significant even after all other predictors are taken into account; its information therefore is largely unique and additive to that of the other predictors.

**Table 4:** Multivariate CPH analysis encompassing most variables.

Covariate	b	SE	z	p
Square ratio	-0.311	0.122	-2.556	0.011
Age	0.024	0.010	2.402	0.016
BNP	0.271	0.127	2.138	0.033
β-blockers	-0.636	0.2594	-1.5295	0.039
Etiology	0.5191	0.2818	1.684	0.065
NYHA	0.408	0.228	1.79	0.073
LVEF	-0.034	0.020	-1.703	0.089
25(OH)D	0.359	0.249	1.441	0.150
eGFR	0.008	0.006	1.28	0.200
FGF23	0.125	0.102	1.232	0.220
Previous admission to hospital	0.170	0.241	0.546	0.384
Sex	-0.254	0.296	-0.859	0.390
PTH(1–84)	0.002	0.003	1.03	0.5099
Calcium, total	0.078	0.179	0.439	0.660

Clinical variables and biomarkers with low statistical significance indicate the use of the same predictive information. The high significance of the square ratio indicates contribution of information distinct from the other predictors. The biomarkers with highly skewed distributions were Log-transformed (Square ratio, 25(OH)D, BNP, FGF-23). Square ratio = (1,25(OH)_2_D)^2^/PTH(1–84).

In HF patients with normal kidney function (eGFR>60 mL/min/1.73m^2^; n = 74) the ratios of 1,25(OH)_2_D/PTH(1–84) and (1,25(OH)_2_D)^2^/PTH(1–84) remain predictive of CV death (p<0.01; data not showed). In HF patients with a history of previous admission to hospital the ratios of 1,25(OH)_2_D/PTH(1–84) and (1,25(OH)_2_D)^2^/PTH(1–84) remain predictive of CV death (p<0.03; data not showed).

## Discussion

The main objective of our study was to investigate the prognostic value of 1,25(OH)_2_D, measured with the new generation assay, and its ratios to PTH(1–84) for long-term CV death in patients with chronic HF. These results clearly demonstrate the relationship between decreased levels of 1,25(OH)_2_D and long-term CV death in HF, and most importantly, the novel use of the 1,25(OH)_2_D to PTH(1–84) ratios as superior biomarkers for the prognosis of HF patients.

Vitamin D deficiency was previously shown to be associated with cardiovascular diseases [25;26], however the biologically active metabolite 1,25(OH)_2_D was not routinely assessed due in part to the lack of assays that are accurate and precise at low 1,25(OH)_2_D concentrations. Decreased 1,25(OH)_2_D levels in HF patients were previously reported [25;27]; once again, the concentrations were obtained with less sensitive and reliable assays. The development of a novel automated, extraction-free 1,25(OH)_2_D immunoassay, with precision and sensitivity superior to LC-MS/MS, has allowed the exploration of new biological relationships that until now were beyond the reach of current methodologies [22]. The 1,25(OH)_2_D test used here, in contrast to the tests for existing biomarkers, offers several advantages: automation, standardization, small sample size and short turn-around-time. In addition, the pre-analytical extraction step, on samples routinely in excess of 1 mL that is normally required for other 1,25(OH)_2_D assays LC-MS/MS inclusive, is not required. This greatly diminishes the imprecision of the assay, especially at low concentrations [22]. The effect on imprecision that results from combining values from two assays into a single ratio was also examined by running two different lots each of 1,25(OH)_2_D and PTH(1–84) kits, and determining the ratio for the four resulting kit combinations. The coefficient of variation of the ratio was on average 2.8% for ratios >0.5 with a maximum CV of 6.3% (data not shown).

These data clearly demonstrate the relationship between decreased levels of 1,25(OH)_2_D and long-term CV death in HF. Most importantly these data indicate that the relationship between the 1,25(OH)_2_D and PTH(1–84) hormones afford potent biomarkers for the prognosis of HF patients, as depicted by the Kaplan Meier curves, which disclose markedly early changes in survival according to the 1,25(OH)_2_D to PTH(1–84) ratios. The association of 1,25(OH)_2_D levels and the risk of adverse outcomes in CV diseases was previously observed [25;27], however the 1,25(OH)_2_D to PTH(1–84) ratios are novel biomarkers not previously investigated. The predictive value of 1,25(OH)_2_D to PTH(1–84) ratios were equivalent to established biomarkers of HF severity such as BNP, NT-proBNP and FGF-23 as evidenced by the AUC. These are all the more remarkable given the modest sample size. In contrast, 1,25(OH)_2_D to PTH(1–84) ratios were superior to Gal-3, PTH(1–84) and 25(OH)D. In comparison to the other biomarkers, 25(OH)D levels were not predictive of the outcome. Several studies have related 25(OH)D levels to survival in HF patients [15;16;28]. The differences might be related to a lower median level of 25(OH)D with a high number of HF patients below 15ng/mL but also to length of follow-up which was longer in our study in comparison to the other reports. However, our results are in agreement with some other reports showing that 25(OH)D deficiency was not related to HF in contrast to PTH or FGF-23 [29;30].

In addition, that the 1,25(OH)_2_D to PTH(1–84) ratios were shown to be statistically superior to 1,25(OH)_2_D and PTH(1–84) alone allowed for the integration of interrelated confounders. This now presents the possibility for treatment based on two modulable factors. Though the square ratio appears to be slightly better than the simple ratio, future studies with larger cohorts are needed to determine which of the two ratios will provide the stronger biomarker. The use of squared ratios, like that employed in the calculation of the widely accepted Body Mass Index, is not without precedent [31]. The 1,25(OH)_2_D to PTH(1–84) ratios are potent biomarkers that combine the contribution of the active form of vitamin D, 1,25(OH)_2_D, with circulating PTH(1–84), which was previously shown to be associated with mortality [16;32]. In addition, studies have shown that PTH contributes to the pathophysiology and worsening of HF [14;33]. Secondary hyperparathyroidism was previously observed in patients with untreated and treated HF with reduced left ventricular ejection fraction with second and third generation immunoassays [33–35]. In addition, PTH was shown to have several negative direct and indirect effects on the heart and cardiac cells [18;36]. Furthermore, increased circulating concentrations of PTH might stimulate adrenal aldosterone synthesis, initiating a vicious cycle between hyperparathyroidism and hyperaldosteronism leading to more pro-inflammatory, pro-oxidant and pro-fibrotic actions [37–39].

Like 1,25(OH)_2_D and PTH(1–84), FGF-23 is a key regulator of mineral and phosphorus homeostasis [40;41]. Interestingly, a significant relationship between FGF-23 and PTH was previously documented in chronic kidney disease and HF patients [42–45]. Previous studies have found significantly higher mortality in HF patients with FGF-23 levels >172 RU/mL [45]. FGF-23 and FGF receptors are both expressed in the myocardium, and it was hypothesized that FGF-23 may have a direct effect on the heart and participate in the physiopathology of CV diseases and HF [46;47]. As 1,25(OH)_2_D participates in the regulation of bone and mineral metabolism with PTH and FGF-23, the potential for significant diagnostic interrelations between 1,25(OH)_2_D, PTH and FGF-23 in the physiopathology of the cardio-renal syndrome related to HF is increasing. Our data demonstrate that decreased 1,25(OH)_2_D levels are significantly related to HF severity and to the rise of serum PTH and FGF-23 levels. The decrease of the biologically active 1,25(OH)_2_D concentration**s** according to NYHA classes might be related to the worsening HF and associated related worsening renal failure which can decrease the 1α hydroxylase activity and therefore leading to a reduction of the 1,25(OH)_2_D levels. The fall of 1,25(OH)_2_D might trigger the rise of PTH and FGF-23 contributing to a vicious cycle for cardiovascular function. On the other hand, worsening of HF and renal failure in HF patients leads to secondary hyperparathyroidism and increases of circulating FGF-23, both of which impact the synthesis of 1,25(OH)_2_D. Measurement of the ratios appears therefore as an efficient tool to assess these interrelated players independently of the kidney function.

Moreover, we observed a significant relationship between 1,25(OH)_2_D and CgA (ρ = -0.42, p<0.0001). Like 1,25(OH)_2_D, CgA exerts a crucial role in calcium homeostasis as dense intracellular core granules involving CgA represent the major intracellular calcium reservoir [48;49]. Furthermore, CgA plays a role for in CV diseases and HF [50].

The therapeutic rational for testing 1,25(OH)_2_D and its ratios to PTH in HF patients is that, not only could it enable more reliable risk stratification, it also could serve to guide treatment selection and monitor the efficiency of other medical devices [51]. Previous studies showed that 1,25(OH)_2_D supplementation has protective effects on myocardial fibrosis of diabetic rats [52], that it is effective in preserving endothelial function in hypertension [53], and it improved cardiac function in patients on hemodialysis that had controllable hyperparathyroidism [54]. Indeed, more tailored treatment selection might be guided by aiming for higher 1,25(OH)_2_D/PTH(1–84) ratios. Lower levels of 1,25(OH)_2_D in HF patients could be increased with calcitriol supplementation (or other active metabolite). On the other hand, PTH could be decreased with other pharmacological treatments such as aldosterone antagonists, known to prevent hyperparathyroidism and its consequences [55;56].

In conclusion, based on the present data, 1,25(OH)_2_D to PTH(1–84) ratios are novel biomarkers that strongly and independently predict CV mortality in chronic HF, stronger than 1,25(OH)_2_D alone. In addition, the 1,25(OH)_2_D to PTH(1–84) ratios are comparable or better than currently used biomarkers such as BNP, NT-proBNP, Galectin-3, CgA and FGF-23 for prognosis, but with the potential additional advantage of providing determinants for therapy guidance and patient monitoring.

## Supporting Information

S1 Dataset data set of the studied HF patients. (XLSX)
